# Necrotising colitis related to clozapine? A rare but life threatening side effect

**DOI:** 10.1186/1749-7922-2-21

**Published:** 2007-08-21

**Authors:** Quor M Leong, Kutt S Wong, Dean C Koh

**Affiliations:** 1 Colorectal Surgery Unit, Department of Surgery, Tan Tock Seng Hospital, Singapore; 2 Division of Colorectal Surgery, Department of Surgery, National University Hospital, Singapore

## Abstract

We report here a case of a 34-year-old gentleman who developed right-sided necrotising colitis after clozapine usage. Anticholinergic activity is believed to the cause. We believe that in patients who have been consuming medications known to have an association with necrotising colitis, constipation with concomitant increasing abdominal pain, distension and fever should be treated with a strong index of suspicion. Consideration of necrotising colitis should prompt expeditious resection of the affected colonic segment.

## Background

Several medications are known to cause necrotizing colitis. These include chemotherapeutic agents, antibiotics and hyperosmolar laxatives [1]. On rare occasions, necrotizing colitis has been linked to phenothiazine usage [2]. This is believed to be due to the anticholinergic effects of phenothiazines, especially when used in conjunction with other psychiatric drugs. Clozapine-induced necrotizing colitis has been reported recently in the literature [3]. Although not a phenothiazine, clozapine is known to have anticholinergic activity. We report another case of clozapine-induced necrotizing colitis and consider the probable mechanisms linking proposed cause and effect.

## Case presentation

A 34 year old Chinese gentleman with a 15-year history of chronic schizophrenia was admitted to the surgical department for acute abdominal pain. He had been treated with clozapine (100 mg in the morning and 200 mg in the night), sodium valproate (400 mg three times daily), benztropine (2 mg three times daily) and diazepam (10 mg in the night) for the last 4 months.

A history of abdominal pain, distension and fever for a day was elicited. This was associated with constipation over the preceding week. There were also complaints of nausea and a single episode of vomiting.

Physical examination revealed a distended and mildly tender abdomen, without localizing signs. Rebound tenderness was absent and bowel sounds were sluggish. Hard faeces were noted on rectal examination. He was febrile at 38.2°C, was tachypnoeic, had a systolic BP of 100 mmHg and was clinically dehydrated.

An abdominal radiograph showed large bowel dilatation affecting the entire colon. The diameter of the colon was greater than 10 cm. There was evidence of faecal loading throughout the large bowel. No dilated small bowel shadows were noted. The erect chest radiograph did not reveal any evidence of pneumoperitoneum. The blood results showed a raised total white cell count of 26.3 × 10^9^/L (4–10 × 10^9^/L), haemoglobin level of 15.9 g/dL (13.0–17.0 g/dL) and a platelet count of 238 × 10^9^/L (160–390 × 10^9^/L). Arterial blood gas results were consistent with metabolic acidosis. Serum potassium, sodium and chloride were all normal but the serum creatinine and urea levels were raised at 328 umol/L (55–100 umol/L) and 15.8 mmol/L (2.0–7.5 mmol/L) respectively. Thyroid function tests comprising of free thyroxine and thyroid stimulating hormone levels were normal at 25 pmol/L (10–20 pmol/L) and 2.0 mU/L (0.40–3.98 mU/L) respectively.

Blood cultures were taken and he was resuscitated with intravenous crystalloids. Broad spectrum intravenous antibiotics comprising of a third generation cephalosporin and metronidazole were administered. A nasogastric tube was inserted for decompression. Rectal manual evacuation was attempted but this was unsuccessful in relieving his symptoms. A fleet enema was administered and this was followed by the insertion of a rectal tube. With no improvement in his clinical signs, an endoscopic decompression was attempted by the surgical registrar on call. However, the entire rectum and sigmoid were filled with solid faecal matter which made it impossible to complete the procedure successfully.

Upon review 2 hours later, the abdominal signs remained unchanged and in view of the persisting tachycardia and a low systolic pressure of 90 mmhg, a decision for an emergency laparotomy was made.

## Operative findings

Operative findings during laparotomy were that of a markedly dilated colon extending from the caecum to the proximal transverse colon. The maximum diameter of the large bowel was at the caecum which measured 12 cm. There were patches of serosal ischaemia extending from caecum to transverse colon. The mesenteric vessels were patent with good pulsations. No perforation or soilage was seen.

In view of the haemodynamic lability, the decision was made to perform a right hemicolectomy without primary anastomosis. The terminal ileum was brought out as a Brookes ileostomy and the distal end of the colon was stapled off with an ILA100 stapler and hitched to the ileostomy.

## Pathology

### Gross description

The caecal wall showed irregular areas of necrosis and linear mucosal ulcers with thinning of the wall. Intervening mucosa was oedematous and unhealthy. Microscopically the mucosa showed extensive necrosis with basal regeneration, characteristic of ischaemic damage. The submucosa and muscularis mucosae were largely spared. Examination of the ileal mucosa showed no evidence of necrosis.

### Microscopic description

**Figure 1:**
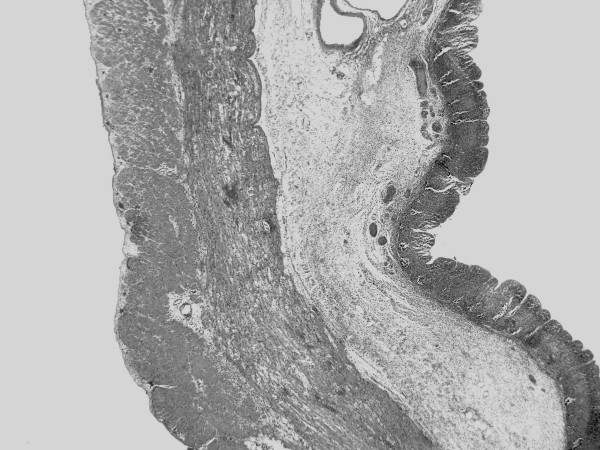
Low power view of colon wall with mucosa, submucosa and muscularis propria. Mucosa shows extensive necrosis while muscularis propria and submucosa are spared (original magnification × 20).

**Figure 2:**
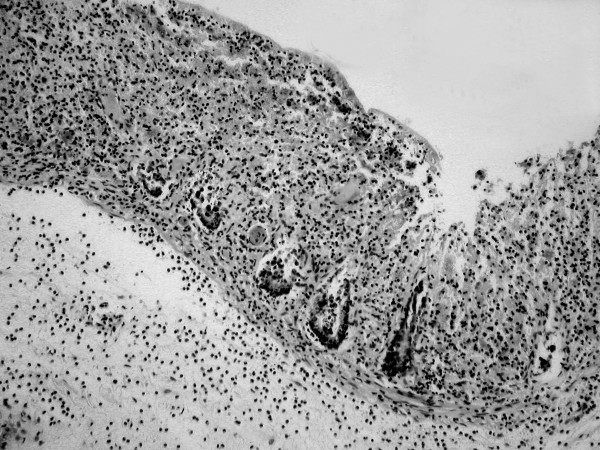
Higher power view of mucosa with muscularis mucosae and superficial submucosa. Crypts are extensively destroyed but are regenerating from the base up, characteristic of ischaemic damage (original magnification × 100).

Sections of the colon showed extensive ischaemic necrosis which affected mainly the mucosa and some focal extension into the muscularis propria (Figure 1 and 2).

### Clinical progress

The patient spent 5 days in the surgical intensive care unit. He required inotropic and ventilatory support initially. These were gradually weaned off successfully. He was extubated on the 5^th ^postoperative day. The rest of his recovery was uneventful and he was discharged on the 14^th ^postoperative day.

Discussion with the in-house psychiatrist resulted in a decision to resume his psychiatric medications. In light of the greater clinical benefits attained with clozapine, the patient was restarted on clozapine upon discharge.

## Discussion

Clozapine, an atypical antipsychotic agent of the dibenzodiazepine class, is characterised by relatively weak central dopaminergic activity as well as anticholinergic properties [4]. It was strongly suspected to be the causative agent in this case.

The term "necrotising colitis" was first used in 1961 to describe a fulminating gangrenous process in which there was no apparent occlusion of the mesocolic vessels [5]. Common ischaemic causes like atherosclerosis, atrial fibrillation and hypotension were excluded. The subject in this case was young, had no cardiovascular problems and the colic vessels were pulsatile during the operation.

Studies have reported that up to 14% of patients on clozapine had gastrointestinal symptoms such as constipation, nausea and vomiting [6].

It is believed that marked anticholinergic gastrointestinal manifestations can lead to necrotising colitis in several ways. Colonic tissue mucosa is particularly vulnerable to intraluminal distension, resulting in decreased capillary circulation [7]. Therefore it is likely that the case patient who had been constipated due to clozapine had prolonged "colonic ileus", leading to colonic dilation. This could have contributed to colonic mucosal ischaemia. A severe state of faecal retention would have resulted in increasing colonic distension, gas, fluid accumulation and bacterial proliferation in the affected segment. The affected ischaemic mucosa would then be a target for invasion by pathogenic bacterial organisms leading to necrosis and systemic sepsis.

It is noteworthy that the right colon was affected both in our patient and the other only reported case in literature. We believe that in the presence of a competent ileocaecal valve, the right colon, particularly the caecum, being the most capacious part of the bowel (Law of Laplace: Tension is proportionate to Radius^4^), bears the brunt of intraluminal distension, resulting in acute mucosal ischaemia and bacterial invasion and subsequent necrosis [8].

## Conclusion

This report serves to highlight a life-threatening side effect of clozapine. It is important that potentially lethal complications like necrotising colitis be ruled out especially when the use of these drugs is associated with unintended non-neuroleptic activity. Constipation, although a common gastrointestinal side effect, must be treated with an index of suspicion, especially when it is prolonged or associated with abdominal pain, distension and fever. When necrotising colitis is strongly suspected, particularly with a history of usage of drugs with a known association, surgical resection of the affected bowel must be performed expeditiously after broad-spectrum antibiotic therapy and fluid resuscitation.

## Authors' contributions

QML attended to the patient, participated in the surgery and drafted the manuscript.

KSW and DCK revised the manuscript critically for important intellectual content.

All authors read and approved the final manuscript.

## References

[B1] Cappel MS, Swan T (1993). Colonic toxicity of administered medication and chemicals. Am J Gastroenterol.

[B2] Hay AM (1978). Association between chlorpromazine therapy and necrotizing colitis: Report of a case. Dis Colon Rectum.

[B3] Shammi CM, Remington G (1997). Clozapine induced necrotizing colitis. J Clin Psychopharmacol.

[B4] Coward DM (1992). General pharmacology of clozapine. Br J Psychiatry.

[B5] Killingback MJ, Lloyd Williams K (1961). Necrotizing colitis. Br J Surg.

[B6] Fitton A, Heel RC (1990). Clozapine: a review of its pharmacological properties, and therapeutic use in schizophrenia. Drugs.

[B7] Boley SJ, Agrawal GP, Warren AR (1969). Pathophysiologic affects of bowel distention on intestinal blood flow. Am J Surg.

[B8] Saegesser F, Sandblom P (1975). Ischemic lesions of the right colon: A complication of obstructive colorectal cancer. Surg.

